# Back to the future – from nuclear energy to AI

**DOI:** 10.1186/s42467-025-00017-y

**Published:** 2025-05-26

**Authors:** Ivo D. Dinov

**Affiliations:** Statistics Online Computational Resource, Departments of Systems, Populations and Leadership, and Computaitonal Medicine and Bioinformatics, and Michigan Center for Applied and Interdisciplinary Mathematics, University of Michigan, Ann Arbor, MI 48109 USA

## Abstract

There are striking parallels between the historic realization that the atomic forces may be harnessed to generate nuclear energy and the recent explosion of research activities and wide proliferation of artificial intelligence (AI). This perspective piece compares and contrasts developments of controlled nuclear fission (CNF) and AI. It also delineates some of the homologies between nuclear energy and AI in terms of the underlying drivers of technological progress, social, economic and environmental challenges, and unimaginable opportunities to transform the course of human civilization. It appears that the course of contemporary AI parallels the trajectory of classical energy generation by nuclear fission; disruptive and unsettling. However, the future prospects of a long-term sustainable NextGen AI may more closely resemble the quest for clean, renewable, and limitless nuclear fusion energy; always pseudorealistic and never decisively resolved.

## Brief summary

The future of AI resembles the quest for clean, renewable, and limitless nuclear fusion energy; always pseudorealistic and never decisively resolved.

The development trajectories of classical nuclear energy generation and contemporary generative artificial intelligence (AI) reveal striking parallels in their origins, progress, and societal implications. Both fields emerged from profound scientific breakthroughs – nuclear energy from the discovery of fission and AI from the formulation of artificial neural networks and large language models – and both rapidly transitioned from theoretical concepts to transformative technologies. Early nuclear power development, epitomized by its role in the Manhattan Project [1] and subsequent civilian applications [2], mirrors the swift rise of generative AI, marked by the advent of deep learning and large language models like generative pretrained transformers (GPT) [3]. Both technologies have seen exponential growth driven by advances in foundational knowledge (e.g., quantum mechanics and statistical learning) and enabling infrastructure (e.g., highly-efficient nuclear reactors and graphics/tensor processing units). However, this rapid advancement has also surfaced complex challenges, including ethical dilemmas, geopolitical tensions, and questions about their long-term sustainability and safety, reflecting their dual-edged nature as tools for both innovation and disruption.

The potential societal transformations brought by nuclear fusion and transformative AI suggest both hope and disquiet. If nuclear fusion achieves energy net positivity, it could herald a new era of sustainable energy abundance, reshaping economies, reducing geopolitical conflicts over resources, and alleviating environmental crises. However, such a paradigm shift also portends unsettling disruptions to existing energy markets, labor systems, and geopolitical stability. Similarly, NextGen AI [4], if realized as a mechanism for augmenting human intelligence, could foster unprecedented creative, scientific, and intellectual breakthroughs. Yet, the intertwining of human and machine cognition could challenge deeply held conceptions of identity, agency, and autonomy, raising existential questions about humanity’s role in its own evolution. In both cases, the promise of profound transformation is tempered by the specter of potential unintended consequences, including inequities, systemic vulnerabilities, potential misuse, and irriversable social, cultural, and biological alterations (Table 1).

**Table 1:** Provenance of nuclear energy

The existence of the smallest building blocks of matter, atoms, was hypothesized by ancient Greek scholars, including Democritus and Leucippus (460-370 BC) [5]. However, contemporary scientific understanding of atomic structure, radiation, and the processes of nuclear fission and fusion matured in only five to six decades starting in the late 1890’s. Although both processes yield enormous amounts of residual energy, nuclear fusion combines two light atomic nuclei into a single heavier atomic nucleus, whereas nuclear fission splits a heavier atom into a pair of lighter nuclei. Constant progress is made to bring fusion to the break-even point beyond which reaction output energy exceeds the supplied input energy. Despite massive research investments, there is still no known viable path to make nuclear fusion a net energy-positive reality [6]. On the other hand, controlled nuclear fission (CNF) has powered nuclear reactors over the past 70 years. Nuclear fission is a chain reaction process where heavy atoms, e.g., uranium, bombarded with neutrons are split apart into lighter isotopes. The result of the heavy atomic nucleus split includes twice or three times as many neutrons, which continue the chain reaction forward. This process releases massive amounts of energy in the form of heat and radiation, and results in some byproducts – lighter radioactive nuclear waste. As more neutrons are also released when the heavier atoms split, the most important step in this fission process is the tight control of the amount of neutrons that propagate the chain reaction forward. CNF is critical to ensure the chain nuclear reaction produces the desired amount of energy, rather than too few neutrinos, fizzling out the propagation, or too many and imploding exponentially as a nuclear bomb.	

Nuclear fusion and transformative AI share an enigmatic and potentially unattainable nature, as they hover on the brink of decisive realization while remaining perpetually incomplete. Fusion has long been an aspirational goal, tantalizingly close but always a few breakthroughs away, and AI, while increasingly capable, continually exposes limitations, overpromisses, and continual morphogenesis of algorithmic comprehension and generalization. Both represent pseudorealistic revolutions – paradigms that transform what is possible but never entirely fulfill their conceptual ideals. As humanity navigates these trajectories, the co-evolution of these technologies with societal structures and other potentially confounding discoveries, e.g., quantum computing, necessitates a balance of optimism, caution and realism. This recognizinges the perpetual interplay between the unlimited human aspiration, the elestic nature of confounding boundaties, and continual adaptation in the face of transformative potential.

History frequently serves as a reflective lens, revealing cyclical patterns and instructive echoes that illuminate plausible trajectories for what lies ahead. In the quest to harness fundamental physical laws to power humanity forward, three distinct phases stand out [7]. Phase one (1890–1935) focused primarily on understanding the basic scientific principles governing the natural world, the development of quantum physics, and the formulation of the matter-energy paradigm. The phase two period (1935–1945) was laser focused on controlling nuclear fission reactions and building atomic weaponry. The post Second World War period (1945–1960) pivoted nuclear advances from bombs to peaceful applications – harnessing of nuclear forces to construct fission reactors, powering submarines, and generating relatively cheap renewable electricity. Over the past half century, nuclear research evolved towards safety, reliability, and sustainability of nuclear power. The next phase of energy generation based on nuclear fission will be focused on robust management (accident prevention) and responsible disposal of the unavoidable and long-lasting radioactive byproducts (nuclear waste) [8]. More specifically, the third phase of nuclear fission developments may be focused on novel ideas for phenomenological modelling, theoretical description of quantifying nuclear fission interactions, developing a comprehensive approach to fission observables, and linking intrinsic-system models and symmetry-conserved observables [9, 10]. Indeed, the holy grail of nuclear physics is the possibility of potentially revolutionary controlled nuclear *fusion*. In contrast to CNF, nuclear fusion releases energy like a solar core dynamo, by merging lighter atoms into heavier ones with much less harmful radioactive isotope byproducts.

In many ways, the evolution of artificial intelligence parallels the long, tortuous, deeply nonlinear, and still-incomplete saga of harnessing nuclear forces to satisfy humanity’s boundless appetite for energy. As early as 300 BC, ancient Greek artisans cast the bronze mythological giant Talos to guard the island of Crete by imaginatively throwing boulders at hypothetically invading ships. Over a millennia later, the Persian scholar Al-Jazari envisioned programmable automata as mechanical devices (1206 AD) [11]. During the Renaissance, Leibniz and Descartes suggested that all rational thought could be made as systematic as algebra and reduced to mechanical calculations [12] (1680’s). Nuclear physics was fully mature when Alan Turing invented the programmable digital computer [13] (1940). This ultimately led to algorithmic machine abstractions of mathematical reasoning and the first AI vision, articulated as a novel concept during the 1955 Dartmouth Summer Research Project on Artificial Intelligence [14]. Unfortunately, at this time, there was insufficient computational or electric power to support the nascent grand AI inspirations. Little progress was made during the AI-winter, which followed for almost half a century. Eventually, an AI-spring started in 1997, when IBM’s Deep Blue beat the reigning world chess champion Garry Kasparov. Over a decade later, an AI-summer (2012) ensued, driven by the proliferation of graphics and tensor processing units (GPU/TPU) augmenting the classical central processing units (CPUs). In earnest, the development, productization, and immersion of foundational generative artificial intelligence models (GAIMs) started in 2022 with the OpenAI release of the first public GAIM, a generative pretrained transformer chat-bot, ChatGPT, which captured mankind imagination by demonstrating a powerful human–machine textual communication.

The inspiring promises and potential perils of both nuclear energy and AI technologies are undeniably boundless. At the one hypothetical extreme, either inappropriate or malicious use of nuclear power or unethical or unintended utilization of AI can wipe out humanity and change the face of the Earth forever. At the other extreme, total and complete risk adversity may at least temporarily (safely) destroy all nuclear weapons and halt all AI developments, at the price of causing significant socioeconomic stagnation. The main challenge for humanity in the century ahead will be to rationalize the known-knowns and known-unknowns, approximate other completely unknown factors, and carefully explicate the expected outcomes corresponding to various energy-utilization and AI-reliance scenarios. There can never be perfect (risk-free, perfect-utility, and high-fidelity) guarantees, so we need to bet on good, rather than idealized impeccable, solutions.

### Drivers

**Fig. 1:**
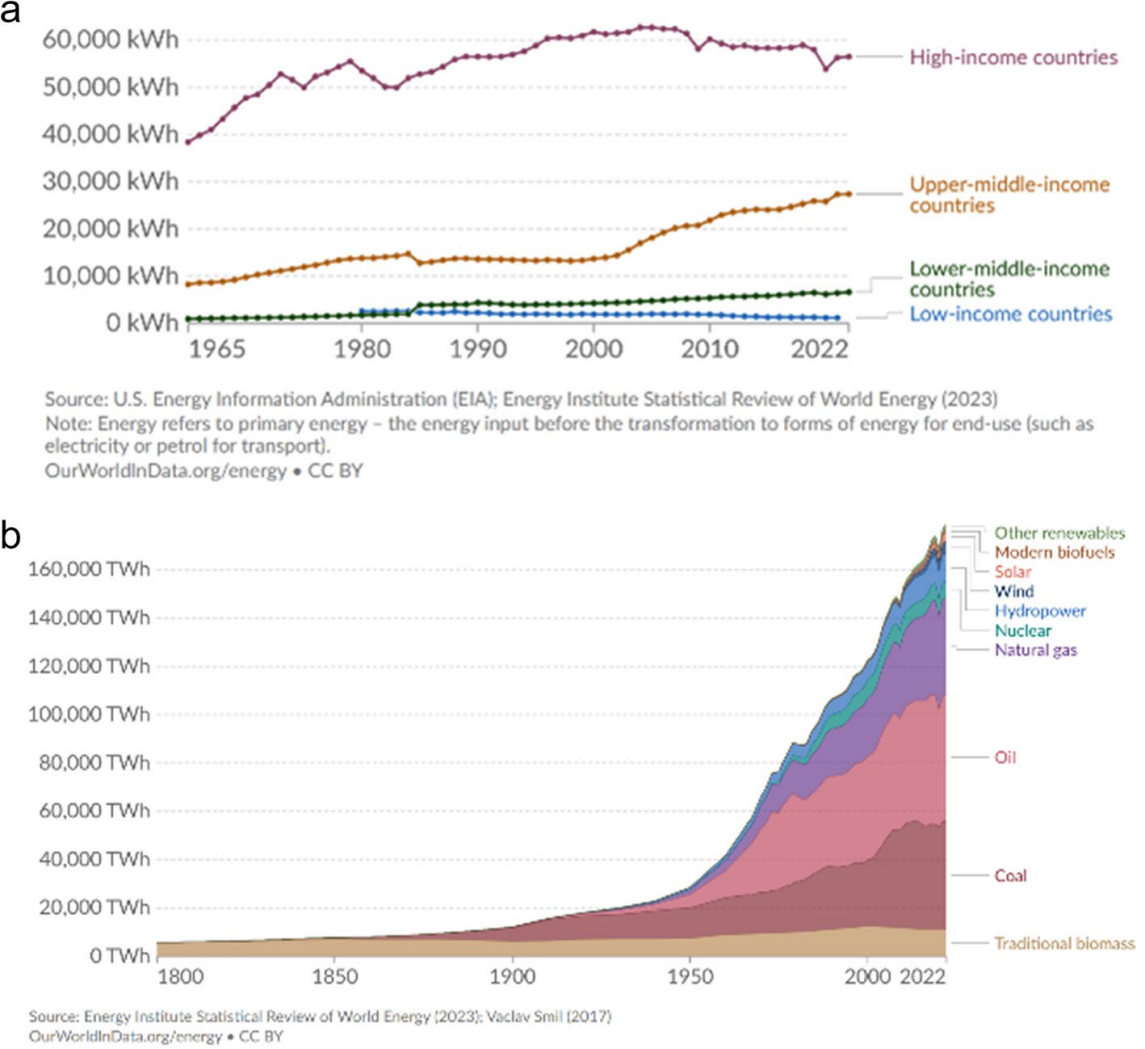
Global energy supply and demand

**Fig. 2:**
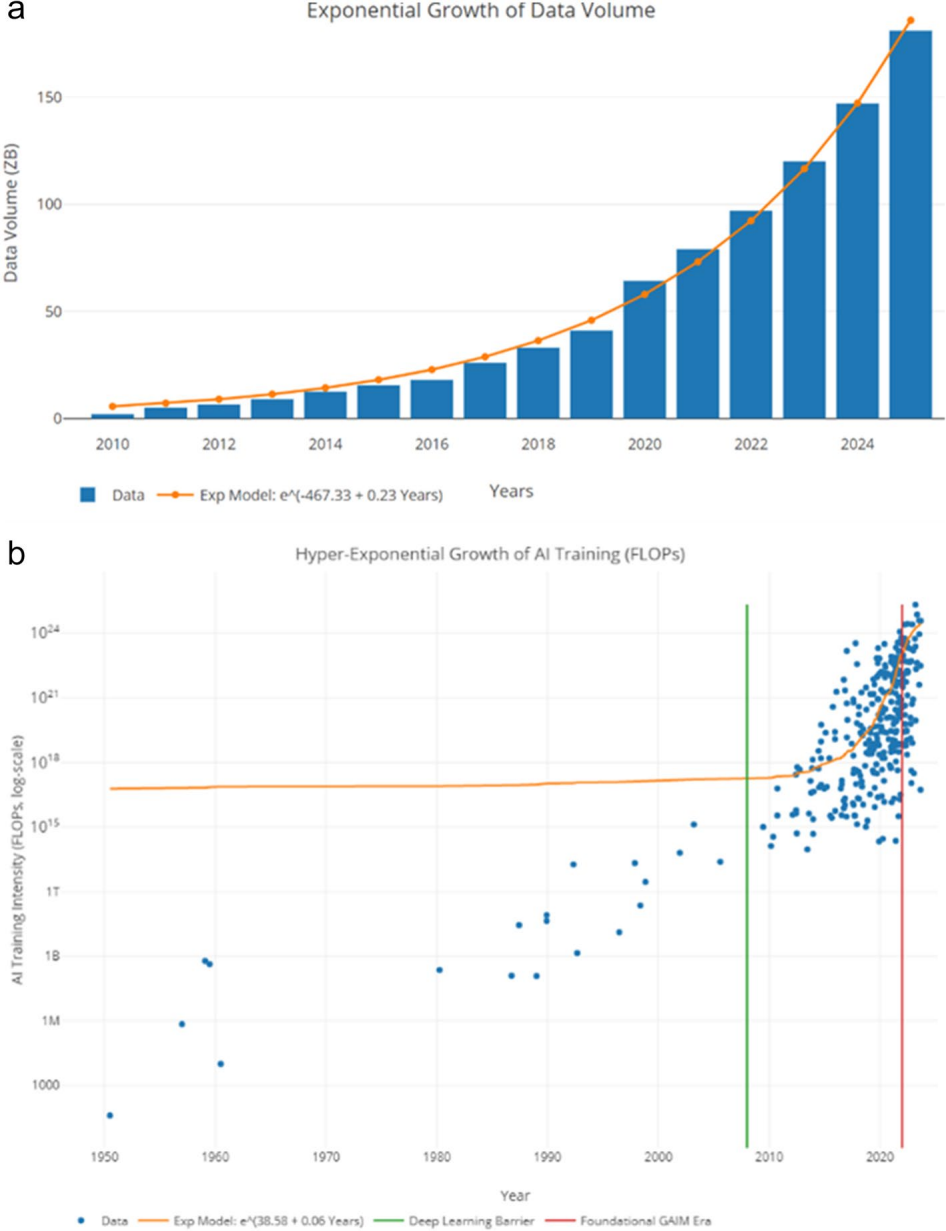
Global nuclear energy consumption (2023)

There are economic, political, social, and environmental reasons to expand nuclear energy production [15], as part of a broader portfolio of renewable energy alternatives reducing the use of fossil-based carbon-fuels. Figure 1 shows globally and across the spectrum of low-, medium- and high-income countries, energy use per capita, which either increased or at best remained steady over the past half century. Extrapolating from the historical human anthropological record (Fig. 1), as life experiences improve across the globe, the energy glut is unlikely to subside in the next century. Unless extreme existential threads plague humanity, both per capita and overall energy demands will probably increase in the foreseeable future. By the end of the twenty-first century, the most likely situation is that the energy needs may double [16, 17]. Without meaningful conservation and sustained energy use reduction (by individuals, within organizations, and across states), the current unilateral focus on transition to renewable energy sources and tunnel-vision decarbonization of energy production may be insufficient to curb the real prospect of severe disruptions to the earth’s climate. It’s clear that carbon emissions and multi-spectral environmental pollution from internal combustion engines powered by fossil fuels represent a real and present danger. What is far from clear is that promoting out-of-sight-out-of-mind strategies for offside classical energy production and supporting ever increasing consumer power overconsumption, coupled with superficial mobility electrification, will alleviate the actual environmental, food supply, human health, and social-justice problems. In many regions, CNF already provides a steady and scalable supply of 20–80% of the local energy demand, Fig. 2. Although construction and maintenance of nuclear power plants is expensive, demands significant supporting infrastructure, has inherent technological limits, and is subject to geopolitical constraints, CNF energy is relatively cost-effective, reliable, and safe. Other drivers of CNF energy production include price-stability, energy-securitization, and global economic vitality. The major risks of nuclear energy generation are associated with unavoidable equipment failures, unpredictable environmental disasters, clandestine maleficent operations, and likely unmitigated incontinence of nuclear waste.

The key determinants driving AI development and utilization closely parallel their nuclear energy counterparts. Various economic, political, social, and environmental reasons influence the massive investment in AI algorithm developments, GPU/TPU hardware innovations, and a wider portfolio of AI applications [18–20]. Among these factors are the need to rapidly increase workers productivity, a holistic push to eliminate most human physical activities, and the strong demand to guarantee tranquil and gratifying human experiences for everyone, anywhere, all the time. With support from appropriate information and communication technologies, AI algorithms are expected to provide instant, efficient, and reliable virtual communication between disparate participants and expeditious decision-making. For instance, distance-based telehealth patient care, completely automated transport and delivery, constant, autonomous and real-time screening, as well as deliberate, thoughtful, and swift decision-making in every situation under any conditions. Another major contributor to the rapid AI proliferation is the ubiquitous digitalization of all human experiences. This reflects measuring, tracking, collecting, analyzing, and learning from observing the complete immersive states of any measurable process. No matter how large (e.g., high spatiotemporal sampling) and complex (e.g., unstructured, high-dimensional, heterogeneous observables) a dataset may be, AI can regurgitate it and provide real-time recommendations, supply rapid process predictions, and compute derived quantities that can inform and support human decision- and policy-making. Many contemporary jobs can be significantly enhanced and optimized by well-trained GAIMs. While initially imperfect, many AI systems are expected to outperform individual human experts and generate better and more consistent outcomes with lower variability, reduced error rates, and diminished costs. In many situations, a major constraint on the broad proliferation of AI is not the overall AI performance, per se, but the breakdown of the authority-responsibility balance with rare yet unavoidable failures. Our cultural values have evolved and aligned with appropriate punitive reactions to human failures. At present, managing AI negligence, mistakes, or biases is difficult. The augmented intelligence social values are not yet well developed. It’s not hard to see why establishing such social norms and deploying augmented human–machine intelligence are believed to be the ultimate goals of building NextGen AI decision support systems.

**Fig. 3:**
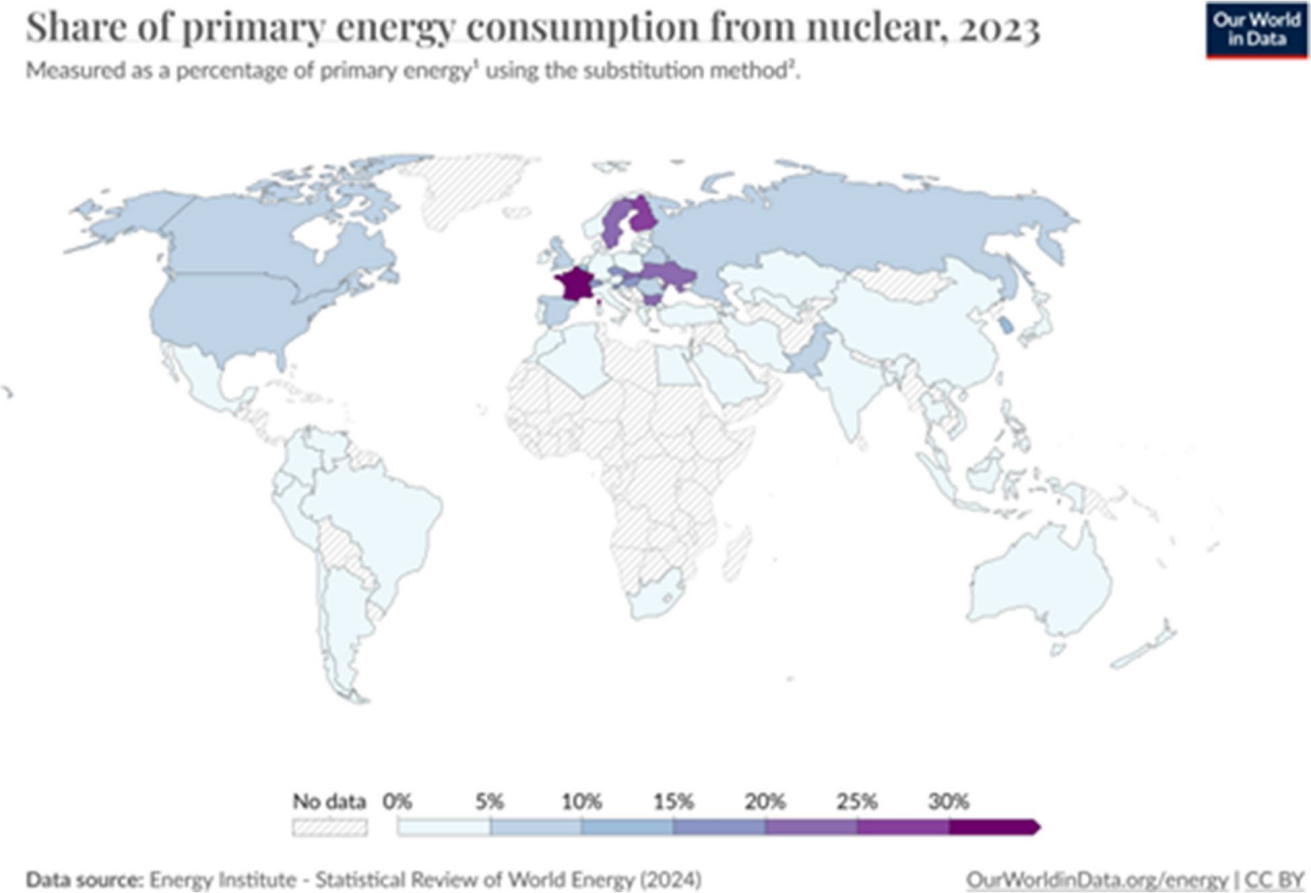
Exponential growth of data driving AI advances

Both data volume and computational power are increasing exponentially, albeit at different rates, see Fig. 3. Kryder’s law states that data volume doubles each 12–14 months, whereas Moore’s law dictates that computing power doubles approximately every 18 months [21], Fig. 3. Within a single lifespan, the genetically hardwired human intelligence is not equipped to evolve at this lightning speed, which reflects the skyrocketing increase of contemporary digital information flow. Think about the evolution of information storage over the past two centuries, from filing cabinets, to punch cards, magnetic media, optical disks, solid-state chips, and Cloud storage. AI offers a viable way to augment human abilities by blending biological Darwinian genetic evolution with data-driven programmatic technological adaptation. The success of this augmented intelligence is predicated on limitless supply of energy, open-scientific communication, unrestricted access to information (to train, refine, and transfer-learn heterogeneous AI systems), and favorable regulatory frameworks.

### Status quo

Currently, CNF satisfies about 11% of the Global electricity demand. Due to an upper limit imposed by the second law of thermodynamics, all heat engine power plants, including energy generation from nuclear fission or burning of fossil fuels, share a similar thermal efficiency. A typical coal or nuclear power plant operates at about 33–37% thermal efficiency. Some marginal improvements in CNF designs may increase this efficiency to no more than 45%, but these marginal gains trade off with risks [22]. Even if we manage to engineer nuclear fusion reactions that are net-energy positive, this upper bound on thermal efficiency will remain as a limiting factor. At the same time, compared to CNF nuclear waste, a hypothetical nuclear fusion reactor may provide other substantive benefits. For instance, a small volume of lightweight atoms could provide enormous amounts of clean fusion energy with relatively less radioactive byproducts, compared to radioactive waste from fusion power plants. Reaction wastes from nuclear fusion and fission are different. The quantity of fusion waste is much larger, but it mostly comprises low-level waste, i.e., mainly tritium and other activation isotopes but no-transuranic elements and without much long-lived isotopes. This benefit of fusion reflects reduced-activation materials and rapid radioactivity decay [23]. For instance, a kilogram of fusion fuel may yield the same energy as 10,000 tons of fossil fuel. Operating a 1GW fusion power station is expected to require one ton of nuclear fuel for the entire year [24]. This represents a massive operational efficiency improvement. For CNF, the major downside of nuclear energy is the management of nuclear waste. Whereas for nuclear fusion, the main drawback is the seeming impossibility of generating the required conditions mimicking the colossal gravity of the sun’s core, maintaining humongous atomic density pressure and temperature of ${10}^{8} {C}^{o}$ degrees.

In its germinal phase of development, AI tracks a similar trajectory as the early efforts in CNF. AI is highly experimentally driven, the realizations of its colossal potentials often downplay any and all perceived hypothetical problems anticipated by skeptics. Much like with the global proliferation of nuclear power, the AI genie is out of the bottle. History suggests that partial control of AI innovation via self-restraint, government regulations, international treaties, and common sense are not expected to be highly successful in the future. Recall our immeasurable, yet futile, efforts over the past half century to reduce the research, proliferation, and risk of nuclear fuel enrichment and ICBM weapon designs, or to improve environmental conditions, protect marine ecosystems, reduce and recycle waste including plastics, or control junk in the Earth’s orbit.

The pursuit of controlled nuclear fusion and the development of generative artificial intelligence (AI) are two highly ambitious human scientific endeavors. Both efforts aim to harness complex, powerful biophysical principles to enhance human experiences. Yet, net-positive nuclear fusion and optimal-AI remain in a perpetual state of evolution and perfection. There are parallels between the developmental phases in nuclear energy production, particularly nuclear fusion, and artificial intelligence, specifically foundational and generative AI. Table 2 shows some of the direct correspondences between the developmental phases of “*pragmatic*” nuclear fusion and “*optimal*” generative artificial intelligence.

**Table 2:** Parallels between nuclear energy and generative artificial intelligence developments

*Dev Phases*	*Nuclear Energy Production*	*Gen-AI*
*Provenance*	● Discovery of radioactive decay in the early twentieth century ● The theoretical foundations laid by many polymaths, e.g., Rontgen, Einstein, Fermi, Szilard, Fermi, and Rutherford	● Turing and McCarthy established the fundamental computing principles ● The concept of artificial intelligence emerged in 1955, ● Theoretical framework for automated machine learning and symbolic reasoning was established
*Early Applications*	● Experiments to commercialize energy from nuclear sources, e.g., the X-ray pedoscope used to find the perfect shoe for every foot ● Early practical application of nuclear theory leading to the creation of nuclear reactors and atomic bombs during World War II ● Transition from theoretical knowledge to practical applications	● Practical AI applications involved rule-based systems and expert systems ● Semi-autonomous AI for computational mathematical modeling and statistical inference, e.g., power analyses, model-based predictions, parametric and non-parametric statistical inference, and quantitative risk estimation ● Partial success of early AI was due to limited computational power, lagging algorithmic complexity, low volumes of data, restricted model flexibility, and prior assumptions
*Inspiration disrupting the status-quo*	● Unlike fission, nuclear fusion promises a cleaner and virtually limitless source of energy ● Achieving controlled fusion remains an elusive goal due to the immense technical challenges involved in containing and sustaining the necessary high-temperature plasma	● Deep neural networks revolutionized AI, e.g., foundational and generative AI models ● Generative adversarial networks (GANs), GPTs, and large language models (LLMs) enabled AI to create realistic images, text, logical argumentation, and even simulate human-like behaviors ● Truly autonomous, safe, effective, trustworthy, unbiased, and generalizable AI remain enigmatic
*Implementation & Computational Demands*	● Progress in nuclear fusion and genAI depend heavily on computational power, e.g., for simulating plasma behavior in fusion reactors and for training complex AI models	
	● GenAI training and nuclear fusion modeling are predicated on complex designs, pragmatic data collection, and availability of large (heterogeneous and representative) datasets (from experimental reactors, wide scope observable phenomena, and advanced simulation)	
	● Material science breakthroughs are needed for containment vessels withstanding extreme nuclear fusion conditions	● GenAI advancements demand the development of highly specialized processors like GPUs and TPUs
*Challenges*	● Both domains face significant social challenges, e.g., environmental pollution and health safety concerns associated with nuclear energy and ethical, privacy, and socio-cultural concerns surrounding AI ● Building public trust through transparency and regulation is essential for the advancement of both technologies	
	● Nuclear fusion research requires massive investments with uncertain timelines for practical returns	● Generative AI development demands significant resources (e.g., energy, rare metals) ● Rapidly evolving and stochastic market dynamics and stiff competition
	● Nuclear fusion promises a cleaner alternative to fossil fuels, potentially mitigating climate change ● There are issues with disposal of residual nuclear waste	● AI has potential to optimize energy usage across various sectors, contributing to environmental sustainability ● Environmental cost (cf. energy) for training large AI models is unlimited
*Opportunities*	● The ultimate promise of nuclear fusion and generative AI lies in their ability to enable future innovations that are currently beyond our imagination, e.g., fusion energy could lead to new propulsion systems for space exploration, AI could unlock new creative paradigms	
	● Controlled nuclear fusion could revolutionize energy production, providing a virtually limitless and clean energy source ● Fusion energy could democratize energy access and reduce geopolitical tensions over energy resources	● Reliable and responsible AI may revolutionize all sectors of the economy by automating complex tasks, enhancing decision-making, creating new forms of human–computer interaction, and freeing time for alternative human experiences ● AI could democratize access to knowledge and enhance all human capabilities

The above synergies illustrate that both nuclear energy and generative AI trace parallel arcs from foundational scientific discoveries to potentially transformative – yet still elusive – breakthroughs. Early atomic theory and radioactive decay paved the way for nuclear fission, mirroring how pioneers like Turing and McCarthy built AI’s theoretical underpinnings. Constrained by technological limitations and public apprehension, both fields initially produced significant, if incomplete, applications (fission reactors and rudimentary expert systems). Today, the race for nuclear fusion parallels the ascent of deep learning and foundational AI modeling, each demanding vast computational power, advanced materials, and massive investments. Environmental concerns over nuclear waste and fusion’s high-temperature plasma containment find echoes in AI’s ethical, social, and privacy challenges, as both technologies face skepticism and require careful regulation and guardrails to build and sustain public trust. Yet their potentials – clean, virtually limitless energy from fusion and AI-driven innovation across every sector – promise to reshape humanity’s future if these powerful tools are harnessed responsibly and sustainably.

### Future

Many experts agree that modern nuclear fission, and any prospective nuclear fusion technologies, are very likely to play a vital role in sustainably meeting the global energy needs. However, there is a stunning realization that releasing energy by atomic restructuring, splitting heavier atoms or merging lighter atoms by neutron bombardment, always yields radioactive byproducts. To maintain the smooth operation of the continuous nuclear chain reactions, these extraneous waste isotopes have to be periodically extracted, transported, and properly disposed of. Neutron activations also contaminate non-structural components inside nuclear reactors. The residual radioactivity levels of the waste from nuclear fusion energy generators are expected to be much smaller than those of contemporary CNF. However, the volume and mass of fusion waste would be many times larger than that of controlled fission plants with similar electric power outputs. This is secondary to the irreducible on-site power drain, i.e., the proportion of fusion power generated solely to continuously maintain the perpetual nuclear reaction. This baseline electrical demand of nuclear fusion power stations is immutable, whether or not the plant is actively generating electricity. On average, this power drain amounts to about 20% of the aggregate generated fusion electrical power. While CNF is likely to progress in its current steady state over the next several decades, the future of nuclear fusion is highly uncertain, both in terms of its theoretical viability, as well as in terms of its practical utility as clean, renewable, safe, and sustainable energy.

Similar uncertainties obfuscate the eventuality of the current AI activities and any prospective NextGen-AI systems, which may include new cognitive architectures, cumulative learning, ampliative reasoning, episodic learning, meta-reasoning, active perception, and proactive AI self-modeling and self-regeneration. There are more unknowns than known mechanistic, biosocial, political, and environmental factors controlling the AI evolution. Similar to most other scientific discoveries, AI technological advances are bound to be exceedingly non-linear, lopsided, and disruptive. At present, probabilistic inference remains our most effective tool for broadly quantifying and analyzing the risk–benefit balance associated with various potential AI trajectories over the coming century of progressive advancements. Much like with nuclear fusion energy, it’s difficult to specify exactly an optimal AI evolutionary path forward with any certainty. In fact, there will be many alternative foundational AI models and different pragmatic AI developments that balance the enormous utility of AI with any potential existential risks. The value of AI to alter human experiences is undeniable. The central question is whether the benefits of AI augmentation truly outweigh the inherent risks of eliminating certain core human functions and virtualizing most other experiences. Relinquishing significant aspects of human control will likely elicit both excitement and fear. Because humanity remains constrained by slow Darwinian genetic evolution, coping with the exponential expansion of information and accelerating AI progress may prove impossible without profound, systematic social restructuring. Such transformation will require thoughtful societal adaptations and a sweeping realignment of core cultural values.

Nuclear fusion, once overshadowed by the perils of fission, is inching closer to commercial viability thanks to recent breakthroughs. Should it realize its promise of clean, virtually limitless energy, the entire landscape of human enterprise could be transformed. Freed from the constraints of scarce resources, AI models and quantum computing systems would have the unprecedented ability to scale – fueling exponential growth across industry, research, and defense. Yet that very synergy of abundant power, high-performance AI, and quantum processing also brings monumental risks, threatening to surpass our current capacities for oversight and governance.

Generative AI models (GAIMs) are already overturning expectations in science, medicine, and creative endeavors, accelerating discovery and challenging notions of human ingenuity. Coupled with the immense computational potential of quantum computing, future GAIMs could solve seemingly intractable problems – from climate modeling and epidemiology to cryptography – in a fraction of the time. But this “lethal trio” of fusion energy, GAIMs, and quantum computing can just as easily destabilize global financial systems, spark new arms races, or erode individual autonomy if left unregulated.

Table 3 outlines the most significant similarities and differences between contemporary nuclear fission and today’s GAIM functionality. It also offers concrete 50-year forecasts for potential nuclear fusion and NextGen AI developments.

**Table 3:** Past, present (2025), and future (2075) synergies between nuclear energy & gen AI

Nuclear Fission vs. Current GAIMS	**Aspect**	**Nuclear Fission**	**Current GAIMs**
	**Foundational Breakthrough**	Discovery of nuclear chain reactions (1930 s-1940 s)	Deep learning revolution (2010 s), fueled by big data & GPUs
	**Core Promise**	Large-scale power generation; a push toward cleaner energy vs. fossil fuels	Wide-ranging automation; improved productivity & novel creative outputs
	**Principal Fears**	Catastrophic accidents (e.g., meltdowns), radioactive waste, proliferation risks	Loss of jobs, bias in AI decisions, potential misuse (e.g., misinformation), loss of control
	**Infrastructure & Investment**	Requires massive, centralized facilities and extensive safety measures	Relies on extensive computing resources, specialized hardware, vast datasets, and lots of energy
	**Regulatory Hurdles**	Non-proliferation treaties, strict safety guidelines, waste disposal protocols	Emerging AI governance debates on bias, transparency, data privacy, and accountability
	**Public Perception**	Mixed optimism (clean energy) and fear (nuclear accidents, weapons, pollution)	Excitement about breakthroughs vs. concern over AI ethics, automation, and surveillance
Next 50 Years: Nuclear Fusion vs. NextGen AI	**Aspect**	**Nuclear Fusion**	**NextGen AI**
	**Timeline**	Commercially viable net-positive fusion reactors by 2075, $p\in [0.45, 0.75]$	Steady stream of major leaps forward in generalizable, context-aware and agentic models
	**Key Technological Advances**	Breakthrough reactor designs (tokamaks, stellarators), advanced materials, and higher reactor efficiencies	Integration with quantum computing, vastly reduced energy usage, improved explainability, joint human–machine intelligence
	**Potential Benefits**	Near-limitless clean energy, drastic cut in carbon emissions, reshaped global energy markets	New scientific discoveries, autonomous systems for healthcare, climate modeling, large-scale optimization & decision-support
	**Major Risks**	High development costs, uncertainty in global cooperation, possible proliferation of fusion tech for weaponization,$p\in [0.15, 0.3]$. Heliocentric space pollution of nuclear waste,$p\in [0.0, 0.01]$	Hyper-automation leading to widespread job disruption, displacement, or reorganization, $p\in [0.6, 0.8]$. AI-driven geopolitical tensions, unforeseen ethical dilemmas, energy crises, AI-self regeneration,$p\in [0.2, 0.4]$
	**Impact on Society**	Fundamental realignment of energy economics; reduced reliance on fossil fuels; new geopolitical power structures	Transformation of economic models, education, logistics, and creativity; potential for profound shifts in human–machine collaboration
	**Governance & Policy Needs**	Global collaboration on fusion R&D; environmental protocols; robust regulatory frameworks to protect innovation & prevent misuse,$p\in [0.4, 0.6]$	International AI transparency standards; ethical guardrails; equitable resource distribution

### Danger zone

Fully realizing this technological trifecta for the *common greater good* demands an ambitious framework of checks and balances – akin to global nuclear accords but adapted to the age of algorithmic and quantum power. Transparent oversight, equitable access, and robust ethical guardrails must evolve alongside these innovations. If humanity can chart a course that harnesses fusion’s abundance, AI’s transformative creativity, and quantum computing’s raw power in a balanced and rational manner, we stand on the cusp of an era defined by breathtaking achievements. Left unchecked, however, these forces risk plunging us into an unpredictable future where substantial disruption, costly systemic failures, and existential threats overshadow the promise of sustainable progress.

Unconditional happily-there-after scenarios are unlikely for either nuclear energy generation or autonomous AI decision-support systems. In popular culture, nuclear energy swings between two polar opposites, from “*too cheap to meter*” and “*infinitely safer than eating*” to an “*uncontrollable*” demon. Going forward, multiple doomsday scenarios are possible. More fluid cyclical oscillations of overhyped promises and dreary prognostications are ever more likely. During the last century, the threat of nuclear war transformed the former League of Nations, a global community formed after WWI, to the contemporary stronger and more resilient United Nations (1945). In the age of AI, to maintain international order, peace, and security and to ensure environmental sustainability will require a similar all-hands-on-deck approach. There is no silver-bullet to achieve this. The odds of success may nevertheless be improved by rational thinking and by building international cooperation addressing a spectrum of economic, social, humanitarian, cultural, and existential disruptions, which should be expected with the ubiquitous immersion of augmented intelligence and availability of unlimited nuclear energy.

Some potential drawbacks of runaway NextGen AI include profound and entrenched imbalances in our delicate physical-mental-spiritual equilibrium, rapid shifts in core cultural values, an increased prevalence of lethargic tendencies, and an overarching dominance of the virtual over the corporeal. Moreover, the massive proliferation of AI risks overemphasizing hyper-consumption and immediate sensory gratification. Coupled with limited access to vital resources – such as energy – these indulgences may undermine traditionally humanistic pursuits, including constructive introspection, creative ideation, serendipitous discovery, spontaneity, and authentic social engagement.

Throughout our history, humanity has continually oscillated between strong individualistic idealism and distinctly outlined collectivism [25–27]. The evidence spans imperial conquests, cultural preservation movements, independence struggles, moonshot initiatives, space exploration, socioeconomic upheavals, quests for knowledge or power, and the optimization of resource access. Although no one can foresee precisely how artificial intelligence will evolve, our collective past, the current nuclear fission paradigm, and the prospects of nuclear fusion offer substantial clues about what we might expect from future NextGen AI services. Nuclear energy and AI are deeply intertwined, with each affecting the other and possessing the capacity to reshape humanity’s trajectory. Access to vast amounts of energy and rapid technological advances underpin nearly all human endeavors, ensuring that nuclear power and AI will remain integral to a sustainable future. The primary question is how to harness the benefits of nuclear energy and fully realize AI’s potential while safeguarding human culture, protecting terrestrial ecosystems, and preserving the diversity of global flora and fauna.

## Data Availability

No datasets were generated or analysed during the current study.
